# Supplementary material for: BE4max and AncBE4max Are Efficient in Germline Conversion of C:G to T:A Base Pairs in Zebrafish
Source: Cells. 2020 Jul 14;9(7):1690. doi: 10.3390/cells9071690 (PMC7407168; doi:10.3390/cells9071690)

Figure S1

A *twist2* AncBE4max_off target #1 (1/82 embryos)

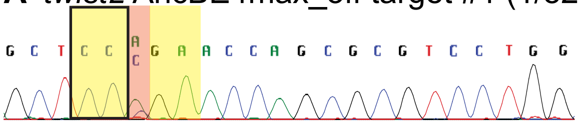

B *twist2* AncBE4max_off target #2 (1/82 embryos)

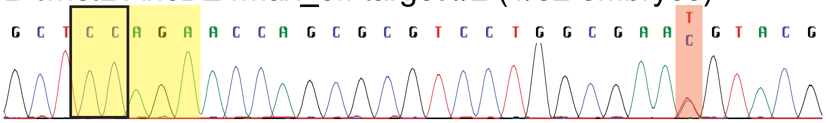

C *twist2* AncBE4max_off target #3 (1/82 embryos)

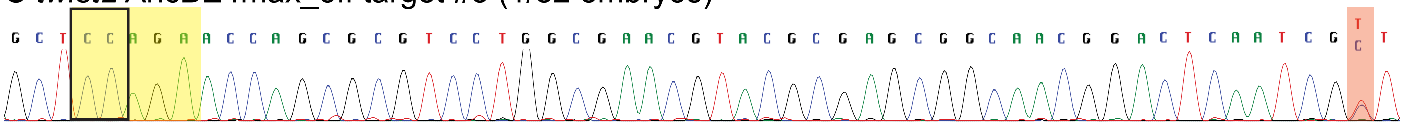

Supplement: Supplementary file 1 [file cells-09-01690-s001.pdf]
